# Present and Potential Future Distribution of Common Vampire Bats in the Americas and the Associated Risk to Cattle

**DOI:** 10.1371/journal.pone.0042466

**Published:** 2012-08-10

**Authors:** Dana N. Lee, Monica Papeş, Ronald A. Van Den Bussche

**Affiliations:** Department of Zoology, Oklahoma State University, Stillwater, Oklahoma, United States of America; University of Regina, Canada

## Abstract

Success of the cattle industry in Latin America is impeded by the common vampire bat, *Desmodus rotundus*, through decreases in milk production and mass gain and increased risk of secondary infection and rabies. We used ecological niche modeling to predict the current potential distribution of *D. rotundus* and the future distribution of the species for the years 2030, 2050, and 2080 based on the A2, A1B, and B1 climate scenarios from the Intergovernmental Panel on Climate Change. We then combined the present day potential distribution with cattle density estimates to identify areas where cattle are at higher risk for the negative impacts due to *D. rotundus*. We evaluated our risk prediction by plotting 17 documented outbreaks of cattle rabies. Our results indicated highly suitable habitat for *D. rotundus* occurs throughout most of Mexico and Central America as well as portions of Venezuela, Guyana, the Brazilian highlands, western Ecuador, northern Argentina, and east of the Andes in Peru, Bolivia, and Paraguay. With future climate projections suitable habitat for *D. rotundus* is predicted in these same areas and additional areas in French Guyana, Suriname, Venezuela and Columbia; however *D. rotundus* are not likely to expand into the U.S. because of inadequate ‘temperature seasonality.’ Areas with large portions of cattle at risk include Mexico, Central America, Paraguay, and Brazil. Twelve of 17 documented cattle rabies outbreaks were represented in regions predicted at risk. Our present day and future predictions can help authorities focus rabies prevention efforts and inform cattle ranchers which areas are at an increased risk of cattle rabies because it has suitable habitat for *D. rotundus*.

## Introduction

Since the introduction of domestic livestock into the New World, vampire bat-transmitted rabies has been the primary disease problem in livestock [1], and *Desmodus rotundus*, the common vampire bat, has served as a major constraint to the success of the cattle industry [2], [3]. *D. rotundus* can feed from the blood of any mammal, but readily feeds on cattle [4], [5], primarily because cattle are a more predictable prey source than wildlife [3]. *D. rotundus* have been reported to roost near a herd and feed repeatedly [1]. In areas with high bat density, a single individual has received 12 bites in one night and had up to four bats feeding at a time [6]. Cattle attempt to shake the bat off, but this is only a temporary reprieve.

Nightly attacks by *D. rotundus* can negatively impact the health of cattle by causing a decrease in mass gain, decreased milk production, increased secondary bacterial infections, and increased risk of rabies or other diseases [3], [7], [8]. In addition to the initial volume of blood loss, the anticoagulant secreted in the saliva of *D. rotundus* causes blood to seep from the wound for hours after the initial bite [9]. Schmidt and Badger [10] reported that cattle owners estimated frequent biting could reduce the amount of milk produced by a single cow 260 L per year and decrease meat production of an individual 39.7 kg per year. Thompson et al. [11] found cattle from typical tropical regions that were in poor condition had a significant increase in milk production when they were injected with an anticoagulant and thus mitigated the negative effects of *D. rotundus*. They concluded that cattle in these areas experience other sources of stress such as extreme climate, inadequate diet, and other parasites, therefore protection from *D. rotundus* is critical. However, an empirical study in Columbia did not find a correlation between the number of vampire bat bites and milk production [12]. There is still not a consensus on the effects of blood loss on cattle.

Nightly parasitism potentially affects meat and milk production, but the primary limiting factor for livestock production throughout Latin America is vampire bat-transmitted rabies [3], [2]. In 1968, over 500,000 cattle died from bat–transmitted rabies in Latin America [13]. With the initiation of bat control methods and vaccines for cattle, these numbers declined to 9,904 reported cases in 1983 [14], 1,831 in 1993 [15], 6,088 in 2000 [16], 3,327 in 2002 [8], and 1,580 in 2006 [15], [17]. While the numbers of reported rabies fatalities have decreased, these are only conservative estimates. The scarcity of diagnostic labs impedes testing of most cattle found dead in the field, suggesting the actual rate of mortality due to rabies is higher [14]. Milk and meat from an animal infected with rabies may still contain the virus, but fortunately, pasteurization and cooking meat to proper temperatures kills the virus [18]–[20]. To date there has been no documentation of a human rabies case resulting from livestock in the U.S. [21].

It is hard to estimate the impact of *D. rotundus* on the cattle industry due to a lack of accurate reporting, particularly in rural areas [2], [8], [22] but Acha and Alba [14] estimated *D. rotundus* were responsible for losses greater than $40 million US during 1983 and again in 1984. These losses, coupled with costs of various preventive measures, can be a significant economic problem for the 18 countries affected by bovine rabies in Latin America [22]. Due to the large expense of controlling the spread of bovine rabies and mitigating the production losses caused by *D. rotundus*, the most effective course of action would be for countries to focus efforts on areas within Latin America where large numbers of cattle and *D. rotundus* co-occur. However, it is difficult to detect such regional locations because the potential area for overlap is too great [16]. An effective way to predict distributions is through modeling species' ecological niches [23]. This method detects associations between environmental variables [in the form of Geographic Information Systems (GIS) layers] and localities of known occurrences of species to generate a probability of the species presence in each pixel of the study area. These predictions can then be plotted on a digital map using GIS software. One specific use of niche modeling is to identify potential areas for disease transmission by highlighting areas environmentally suitable for both the host and vector species [24], [25]. Thus far, ecological niche modeling has been used to predict possible areas at risk for outbreaks of anthrax [26], dengue fever [27], chagas disease [28], chytridmycosis [29], plague [30], and hemorrhagic fever caused by filoviruses [31].

Given the estimate of 70 million cattle at risk in areas where rabies has been reported in the past 10 years [14], [32], we believe ecological niche modeling could be a beneficial tool to predict areas where cattle could potentially have a greater risk of rabies and other negative effects of *D. rotundus*. Cattle rabies occurrences appear to be linked to seasonal climate variation and an increase in bat population size [33], therefore we generated an environmental suitability map for *D. rotundus* and used a published data set of predicted cattle density to indicate areas that may have a higher relative risk of common vampire bat predation or suitable conditions for cattle rabies outbreaks. As cattle density has already been shown to be an important factor to explain the spatial clustering pattern of *D. rotundus* [34], we hypothesize areas with a high density of cattle and suitable environmental conditions for *D. rotundus* could suffer the greatest effects of both nightly parasitism and risk for rabies. We also investigated if the distribution of *D. rotundus* would change and possibly extend into currently unsuitable areas, including the United States, with future climate predictions. Climate change has already been predicted to impact the distribution of European bats [35] and *D. rotundus* in Mexico [36]. The change in amount of suitable habitat may introduce bat predation on cattle not currently affected; however our results do not account for future cattle distributions.

## Methods

### *Desmodus rotundus* predicted potential distribution

To generate the present day potential distribution map for *D. rotundus*, the study area was delimited using the known species' distribution from Mexico south through Central America to Uruguay, Argentina, and Chile, specifically from 28°N to 33°S [37]. There are no known occurrences of *D. rotundus* on Baja peninsula (Mexico) or in the Caribbean islands, except Trinidad, Tobago, and Margarita Island, so these areas were excluded from the present day prediction. Museum records of *D. rotundus* (9,741) were downloaded from the Global Biodiversity Information Facility [38] (http://www.gbif.org/). This organization serves as a data portal to allow free access of information about natural history museum holdings. Occurrence data for *D. rotundus* collected before 1940 were removed because GIS environmental data are not available for that time frame. Records lacking latitude and longitude coordinates were georeferenced in GEOlocate v. 3.22 [39] (http://www.museum.tulane.edu/geolocate/). This web application uses textual descriptions of specimen collecting localities to assign latitude and longitude coordinates to specimens. Depending upon the detail for the collecting locality, the georeferences were assigned low, medium, or high confidence based on the geographic extent of the error associated with the georeference. Records with medium or high confidence scores were included in the occurrence data set. All points were plotted in ArcMap10 [40] to confirm that georeferenced localities correspond with original descriptions. Finally, duplicate records were removed, leaving 984 spatially unique occurrence points for *D. rotundus*.

GIS climatic layers representing minimum and maximum temperature, and precipitation, averaged over the last five decades (1950–2000, hereafter “present”), were obtained from the data portal of the Research Program on Climate Change, Agriculture and Food Security of the Consultative Group on International Agricultural Research [41] (http://www.ccasfs-climate.org/). To predict the distribution of *D. rotundus* in future climates, we downloaded from the same source climate model data (temperature and precipitation) for 2021–2040 (hereafter 2030), 2041–2060 (hereafter 2050), and 2071–2090 (hereafter 2080), downscaled from MIROC 3.2 General Circulation Model (GCM), one of the GCMs used in the Fourth Assessment Report of the Intergovernmental Panel on Climate Change (IPCC) [42]. We used the A2, A1B, and B1 emission scenarios included in the IPCC Special Report on Emission Scenarios. In the A2 scenario, the focus is on regional economic development and slow change towards cleaner technology. It is also characterized by an increase of CO_2_ concentration to 1250 ppm and temperature by 3.4°C in 2100. The A1B scenario represents current trends in which human energy use continues to increase (not relying on one particular energy source), but CO_2_ emissions are stabilized to some degree by technological advances and public awareness. An estimated CO_2_ concentration of 850 ppm and temperature increase of 2.8°C is used. In the B1 scenario, the human population peaks and starts to decline around 2050. There is a switch to using cleaner technology, CO_2_ concentrations increase to 600 ppm, and temperature rises by 1.8°C [43]. Present and future temperature and precipitation variables were used to calculate 19 “bioclimatic variables” for present, 2030, 2050, and 2080 periods representing quarterly and monthly climate seasonality and extremes [44]. Bioclimatic variables were generated in ESRI ArcInfo using available AML code (http://www.worldclim.org/bioclim). All environmental variable layers had a 1 km^2^ resolution and were masked to the extent of the study area in ArcMap10 [40].

Environmental layers and *D. rotundus* occurrences were used in Maxent v.3.3.3k [45], [46] to run the ecological niche models because the maximum entropy algorithm requires presence–only data and has been shown to produce reliable results [47], [48]. Maxent contrasts the environmental conditions associated with presences points with random background points that sample available environmental space where the species could potentially occur. Additionally, Maxent uses “features”, functions derived from the environmental variables, as parameters to keep the model from overfitting the data [46]. We used the auto features option which chooses the features appropriate for the number of occurrences in the data set. Given the relatively large occurrence data set available, we opted for the random seed and test percentage options in Maxent to randomly split the occurrence points (984) into training and testing data sets each with half of the data points (492). Jackknifing was applied for all environmental layers to determine individual percentage of contribution to the model overall accuracy gain. We then ranked the variables by percent contribution. We chose to use the five variables that contributed more than 5% to refine our predictions in a final model. The ecological niche model generated using the subset of environmental variables was projected on the 2030, 2050, and 2080 environmental datasets for each of the three climate scenarios, resulting in nine predictions. Predicting species' distributions using projections of ecological niche models on future climate datasets is an appropriate method for gaining insights to possible changes in species distributions [49], [50], and used to document both range expansions [51]–[53] and reductions [54], [55].

To directly compare present day potential distribution of *D. rotundus* to its potential distribution in 2030, 2050, and 2080, we identified pixels that were unsuitable under present conditions but became suitable in the future predictions. We converted the continuous probability of presence values to a binary output by applying a 10% omission error threshold to the Maxent outputs. This method assigns pixels with a probability of presence value less than the lowest value corresponding to 10% of the training points a value of zero (absent), and pixels with a probability of presence above this value are given a value of one (present). This conversion is more sensitive to “outliers” (locations where the species was collected despite a low predicted probability of suitability) and constrains the pixels initially predicted as present [56]. We were also interested in identifying the environmental variable that most influences the differences between the present day and each future prediction. Maxent v.3.3.3k [45], [46] can address this question by measuring the similarity between present and future climates for each environmental variable. The variable with the largest dissimilarity value for each pixel is then plotted on geographic space [57]. Finally, the present niche model was evaluated for accuracy using the area under the curve (AUC) of the receiver operator characteristic which plots the proportion of presences predicted absent (omission error) against the proportion of area predicted present. An AUC value of 1 indicates a perfect prediction and 0.5 is a prediction no better than random [58]. However, the usefulness ROC AUC to evaluating model accuracy is increasingly questioned [59]–[61]. A clearer but perhaps oversimplified assessment is provided by the omission error alone.

### Cattle at risk prediction

Projected cattle density data for 2005 were obtained from the Food and Agriculture Organization of the United Nations (FAO) [62]. The Animal Production and Health Division of the FAO maintains a public database containing georeferenced data on livestock numbers but these numbers are at different spatial scales for different regions. The FAO used this database with vegetative, geological, environmental, demographic, and climatic variables to interpolate and extrapolate the density of cattle for the world at 1 km^2^ resolution. Pixels in deserts, high mountains, closed canopy forests, and highly urbanized areas were coded as unsuitable habitat for cattle. The resulting prediction can be downloaded from the FAO website (http://www.fao.org/AG/AGAInfo/resources/en/glw/GLW_dens.html) and used as a layer for additional processing in ArcMap10. To highlight the areas with suitable habitat for vampire bats and include a measure of cattle density, the continuous Maxent output for the present day *D. rotundus* distribution was converted to a binary output using a 10% omission error as the minimum threshold value following the methods explained earlier. The cattle density layer was then masked to only show pixels corresponding to predicted presences of *D. rotundus*. Finally, locations for 17 cases of cattle rabies outbreaks reported in the scientific literature or found through ProMED-mail (http://www.promedmail.org) [63], which is a database containing recent alerts on infectious diseases, were plotted on the cattle risk prediction map. We assigned geographic coordinates to each record using GeoNet Names Service online gazetteer (http://geonames.nga.mil/ggmagaz/) and estimated the georeferencing uncertainty based on the geographic extent of the locality description of these outbreaks. We used the georeferencing uncertainty measure to map a zone of uncertainty (GIS buffer) around each outbreak. In some cases, the georeferencing uncertainty was only 2–5 km so we applied a minimum zone of uncertainty of 10 km to all records because Lord [33] reported the majority of bovine rabies outbreaks reach 5–10 km wide. The number of pixels in the uncertainty zone predicted at risk by our model were then calculated. It is important to note some records on ProMED-mail do not report how the cattle acquired rabies, but we assumed vampire bats were the vector.

## Results

### *Desmodus rotundus* predicted distribution

**Figure 1:**
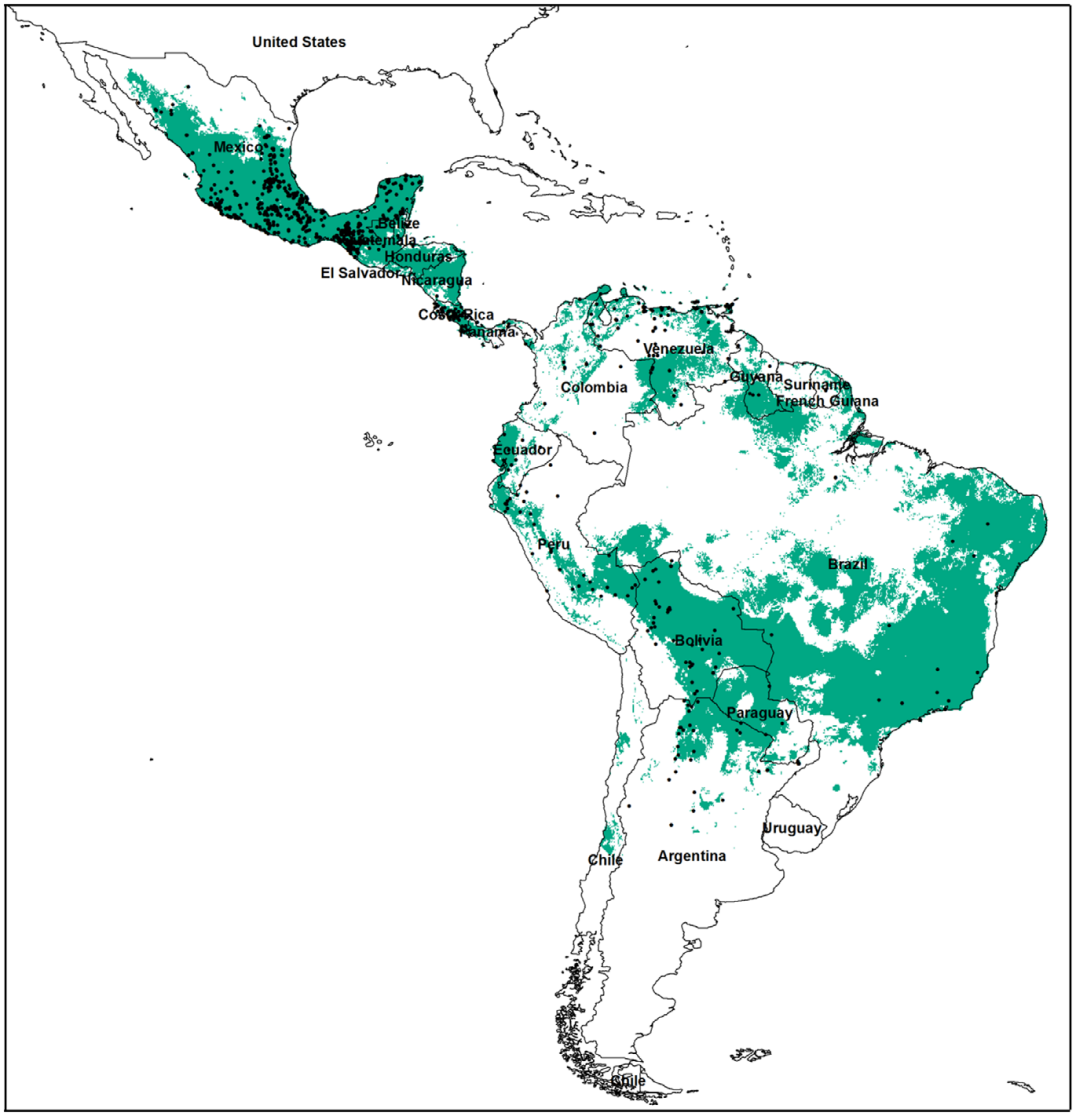
Current potential suitable habitat for the common vampire bat, *Desmodus rotundus*. based on four different environmental data sets. (A) present, (B) 2030, (C) 2050, (D) 2080. Black dots indicate spatially unique known occurrences for *D. rotundus* which were used in model construction.

Five of the climate variables (precipitation seasonality, temperature seasonality, precipitation of the wettest month, precipitation of the driest month, and mean temperature of the coldest month) contributed most to the model (Table 1). These were the variables chosen to include in the final model used to predict the present day and future distributions of *D. rotundus*. A training AUC of 0.826 and a testing AUC of 0.805 indicated the present model performed well using only the top five environmental variables. Our present model predicted most of Mexico and Central America to have suitable environmental conditions for *D. rotundus* (Fig. 1). Other regions of high suitability include portions of Venezuela, Guyana, the Brazilian highlands, western Ecuador, and east of the Andes in Peru, Bolivia, Paraguay, and northern Argentina.

**Table 1:** Jackknife results indicating variable percent contributions to the model.

Environmental Variable	Contribution to first model	Contribution to final model
Precipitation seasonality	28.3	42.4
Temperature seasonality	23.4	24.7
Precipitation of wettest month	11.6	17.5
Precipitation of driest month	7.2	4.8
Mean temperature of coldest month	5.3	10.5
Precipitation of coldest quarter	4.2	
Mean temperature of coldest quarter	4	
Mean temperature of wettest quarter	3.2	
Annual precipitation	2.5	
Precipitation of driest quarter	2.3	
Mean temperature of driest quarter	1.8	
Max temperature of warmest month	1.5	
Temperature annual range	1.1	
Mean diurnal range	1.0	
Mean temperature of warmest quarter	0.8	
Isothermality	0.7	
Precipitation of wettest quarter	0.6	
Annual mean temperature	0.4	
Precipitation of warmest quarter	0.1	

**Figure 2:**
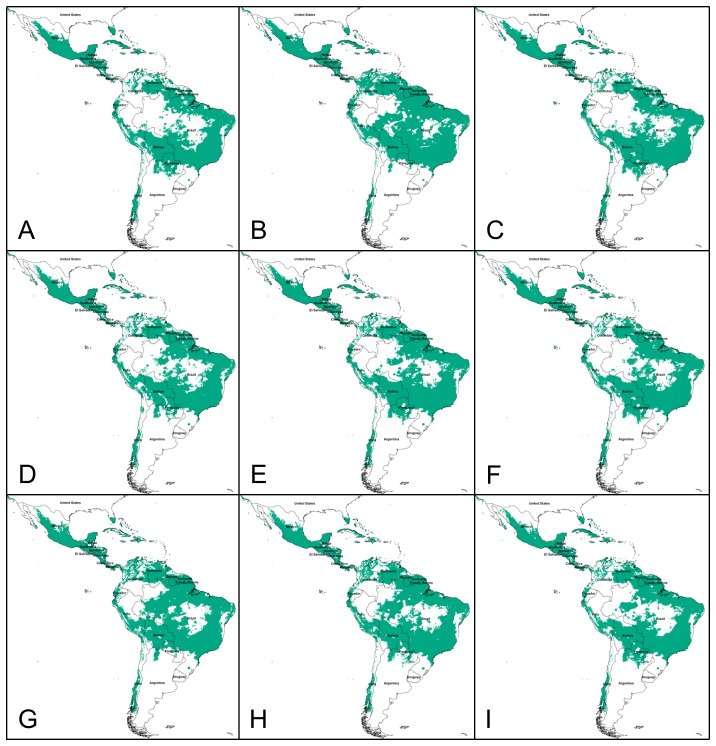
Future potential suitable habitat for the common vampire bat, *Desmodus rotundus*, based on three climate scenarios and time frames. (A) 2030 scenario A2, (B) 2030 scenario A1B, (C) 2030 scenario B1, (D) 2050 scenario A2, (E) 2050 scenario A1B, (F) 2050 scenario B1, (G) 2080 scenario A2, (H) 2080 scenario A1B, (I) 2080 scenario B1.

**Figure 3:**
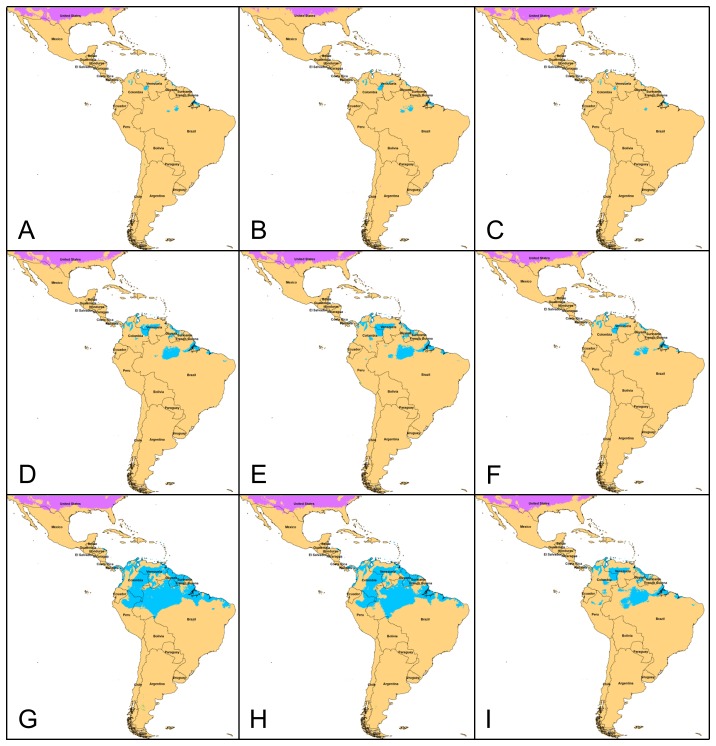
Dissimilarity maps indicating which environmental factor was most dissimilar between present day predictions and nine future predictions. (A) 2030 scenario A2, (B) 2030 scenario A1B, (C) 2030 scenario B1, (D) 2050 scenario A2, (E) 2050 scenario A1B, (F) 2050 scenario B1, (G) 2080 scenario A2, (H) 2080 scenario A1B, (I) 2080 scenario B1. Colored pixels representing dissimilarity between present day and future predictions: Blue for mean temperature of coldest month, purple for temperature seasonality, green for precipitation seasonality, yellow for precipitation of driest month, and pink for precipitation of wettest month.

Generally regions of suitability in the present day models, Mexico, Central America, Venezuela, Guyana, western Ecuador and Peru, and Bolivia, also had high suitability when the model was projected to future climates for 2030, 2050, and 2080 (Fig. 2). Differences among the climate scenarios were most obvious in the amount of suitable habitat for *D. rotundus* in Brazil. There were also areas that would become suitable for *D. rotundus* in the future climates. These included French Guyana, Suriname, and additional portions of Venezuela and Columbia (Fig. 2). The Caribbean region and Florida had suitable habitat for *D. rotundus* under future climates but these regions were not included in the present day model because there are no museum records from these areas. No changes occurred in Central America under future scenarios, and no suitable regions were predicted with any of the future climate scenarios in the U.S., except for southern Florida (Fig. 2). This outcome is in contradiction with previously hypothesized wide range expansion into the U.S. [36], which can be explained by regional differences in ‘temperature seasonality’ of the present day and future climates (Fig. 3). The lack of expansion into new areas in South America could be explained by differences in ‘mean temperature of coldest month’ (Fig. 3).

### Cattle at risk prediction

**Figure 4:**
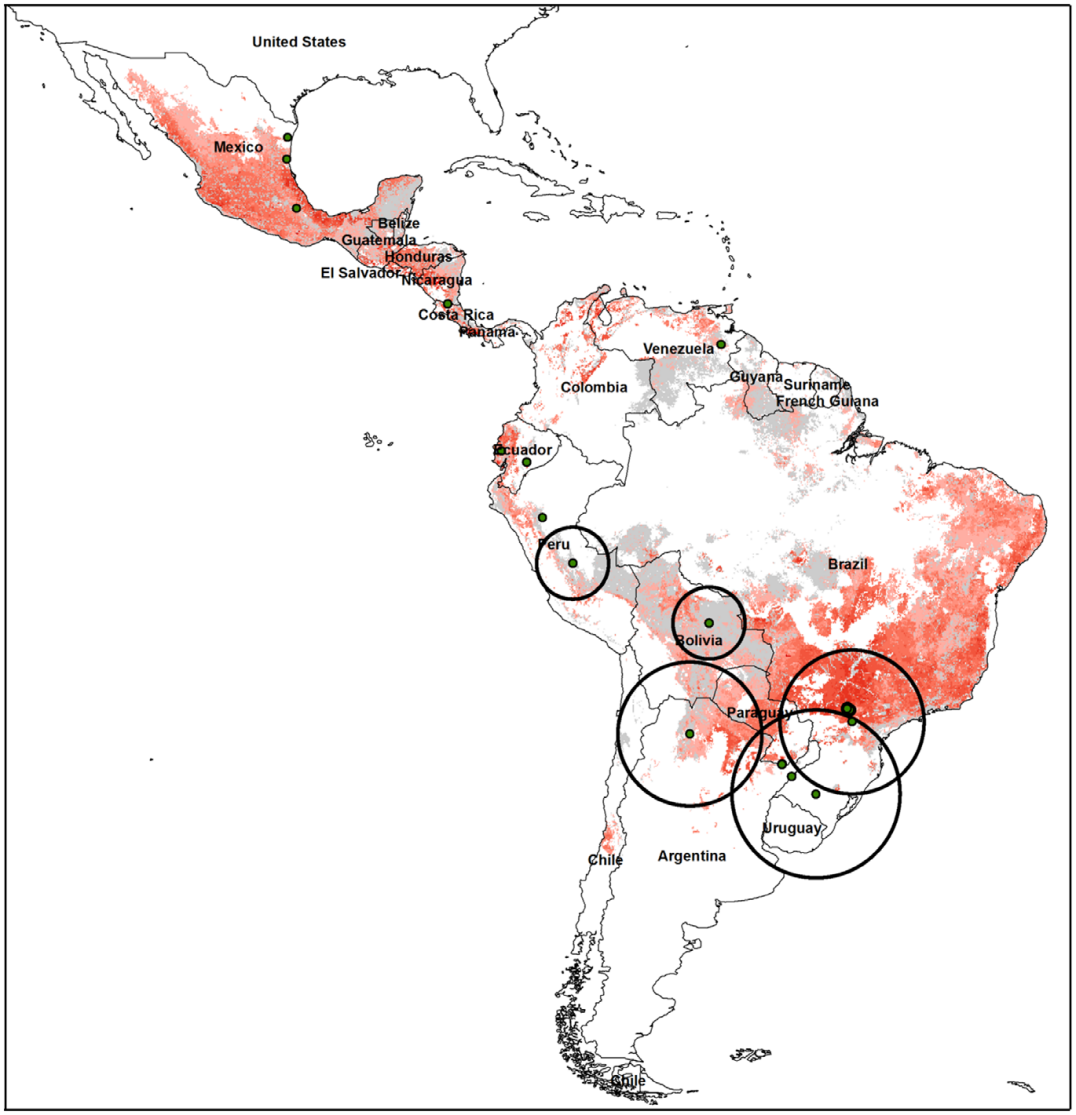
Cattle densities per km^2^ shown in pixels with predicted suitable habitat for the common vampire bat, *Desmodus rotundus*. Cattle density increases with shades of red and gray pixels indicate areas predicted to be unsuitable for cattle by the Food and Agriculture Organization of the United Nations [55]. Green dots indicate documented cattle rabies outbreaks and black circles represent uncertainty zone for each record.

When the 10% omission error threshold was applied to the present day *D. rotundus* distribution, 51.0% of the study area was classified as suitable habitat for vampire bats. This area included most of Central America, which also has a high density of cattle per km^2^, with the exception of the Yucatan Peninsula (Fig. 4). The Yucatan Peninsula was suitable for bats but was classified as unsuitable for cattle in the FAO Animal Production and Health Division cattle data set. The Brazilian highlands also contain land suitable for *D. rotundus* and large numbers of cattle. While the eastern slope of the Andes was predicted to be suitable for *D. rotundus*, most of this mountainous region is not suitable for cattle ranching. The 17 documented cases of cattle rabies were generally in areas where cattle were predicted at risk (Fig. 4). Twelve of the 17 cases had pixels predicted at risk within the georeferencing uncertainty zone (Table 2). Upon closer investigation the five outbreaks with no pixels predicted at risk were in very close proximity to pixels in locations at risk (1–12 km).

**Table 2:** Cattle rabies outbreaks used to evaluate cattle at risk prediction.

Outbreak	Citation	Size of Uncertainty Zone	% “at risk” pixels
Guasipati, Venezuela	[28]	10 km	100%
Olmedo, Manabí, Ecuador	[75]	10 km	100%
Florestópolis, Paraná, Brazil	[75]	42 km	77%
Bela Vista do Paraíso, Paraná, Brazil	[75]	43 km	66%
Aldama, Tamaulipas, Mexico	[56]	10 km	50%
Paraná, Brazil	[75]	656 km	46%
Guarayos, Santa Cruz, Bolivia	[75]	305 km	32%
Salta, Argentina	[28]	680 km	26%
Maltrata, Veracruz, Mexico	[75]	10 km	20%
Rio Grande do Sul, Brazil	[75]	723 km	11%
Oventeni, Atalaya, Ucayali Region, Peru	[75]	320 km	10%
Isla Apipé, Argentina	[28]	27 km	3%
Los Chiles, Alajuela, Costa Rica	[75]	10 km	0%
Rancho Santa Gertrudis, Tamaulipas, Mexico	[75]	10 km	0%
Santo Tomé, Corrientes, Argentina	[75]	10 km	0%
Saposoa, San Martin, Peru	[75]	10 km	0%
Sevilla Don Bosco, Morona-Santiago, Ecuador	[75]	10 km	0%

## Discussion

Our model indicates several environmental characteristics that explain the distribution of *D. rotundus* throughout Mexico, Central and South America. Temperature and precipitation variables are consistent with known ecological requirements of *D. rotundus*. ‘Mean temperature of the coldest month’ and ‘temperature seasonality’ (difference between summer and winter) are among some of the most important predictors of habitat suitability. This agrees with previous research, which suggests the distribution of this species is most limited by the coldest temperature in winter. *D. rotundus* cannot survive in areas that have temperatures below 15°C [13] because thermoregulation in these cold temperatures requires more energy than an individual can consume on a nightly basis [64]. *D. rotundus* also prefers locations with less than 45% humidity [65], which can explain why ‘precipitation of the wettest month’ and ‘precipitation seasonality’ and ‘precipitation of the driest month’ are found to contribute greatly to the model.

Used collectively, these characteristics depict the known distribution of *D. rotundus* well and can be applied to predictions of *D. rotundus*'s distribution in future climates. Two of these environmental variables, ‘mean temperature of the coldest month’ and ‘temperature seasonality’ can be interpreted as limiting factors of *D. rotundus* expansion in South America and the U.S., respectively. Interestingly, different environmental characteristics appear as limiting factors in the two continents, but not surprisingly as multiple variables are required to properly describe a species' fundamental niche.

When the predicted distribution for *D. rotundus* is combined with cattle density data, areas in Mexico, and Central and South America that have cattle with a higher relative risk of harmful effects from vampire bat parasitism are highlighted. Most of Mexico, Central America, Paraguay, and the Brazilian highlands are highly suitable for both *D. rotundus* and cattle. Cattle in this region are likely to be sympatric with *D. rotundus*, suffer from common vampire bat bites, and have a greater risk of contracting rabies. Results from our cattle at risk prediction are not surprising considering Mexico and Brazil are both routinely listed in the top three countries with the most reported cases of cattle rabies [14], [15], [17]. Unfortunately for the cattle industry in Latin America, more land becomes suitable for *D. rotundus* if climate change follows any scenario we used. It is also important to note there is suitable habitat for *D. rotundus* in the Caribbean. With the exception of Trinidad, Tobago, and Margarita Island, there are currently no vampire bats in this region but our results predict they could be successful invaders if cattle are also present. Finally, our model predicts the majority of cattle in the U.S. are safe from the negative impacts of *D. rotundus*, despite global warming trends. While we recognize breeding distributions of North American birds have already moved northward [66], our results suggest *D. rotundus* will be limited by ‘temperature seasonality’ and not expand into the U.S. through Mexico. This result contradicts another report [36] which suggests the Gulf coast of Texas and Louisiana may become capable of invasion. The conlfict in results is based on a difference in environmental variables considered. It seems Mistry and Moreno-Valdez [36] made initial conclusions on the range expansion of vampire bats after examining only a single temperature increase, while this study uses climate scenarios and several climate variables summarizing annual and seasonal temperature and precipitation trends. The agreement between this and the previous report is that southern Baja California, Florida, and the coasts of Mexico could become suitable for the vampire bat with future climates.

As expected, the 17 documented outbreaks of cattle rabies occurred within or nearby areas at risk for harmful effects of *D. rotundus*, suggesting successful utility of our prediction. In addition to the usefulness of our risk prediction, there are other patterns of rabies transmission that could be used in combination with our results to help authorities focus prevention efforts. Epidemiological characteristics of vampire bat transmitted rabies in cattle have been associated with topographical and geographical features [67]. Migration patterns of outbreaks usually follow rivers because there are ample trees for the bats to roost [33], [67]. When strains of cattle rabies isolated from Brazil were examined, groupings of different phylogenetic strains could be explained by elevation boundaries [67]. Also in Brazil, regression analysis indicated cluster patterns of vampire attacks on cattle could be explained by ‘distance to forest’, ‘proportion of sugarcane’, and ‘cattle density’ [34]. In Venezuela, Mexico, and Argentina, the number of outbreaks was correlated with precipitation and the seasonality of vampire reproduction [33]. Currently most countries have scattered efforts that can only respond to areas where cattle have died [33], but considering these factors along with our ecological niche model predictions is critical to mitigating the spread of cattle rabies.

A variety of methods are employed to reduce the harmful effects from *D. rotundus*, including destroying roosts with fire or dynamite [65] or cementing them closed; however these methods also affect any species that cohabitats with *D. rotundus*, such as the threatened Dekeyser's nectar bat, *Lonchophylla dekeyseri* [68]. Alternatively, an anticoagulant poison, diphacinone, is used to kill the bats. When injected into cattle, feeding *D. rotundus* will receive a lethal dose [10], [65], [69], [70]. This treatment is safe on adult cattle but is not recommended on suckling calves [7]. Additionally, the chemical must be routinely injected [71]. Diphacinone can also be mixed with vaseline and placed on a captured bat. After the bat returns to the roosts, this “vampiricide gel” is transferred to colony mates. As the bats then groom themselves they ingest the chemical [70].

As rabies is the most important threat to the cattle, non–lethal methods of bat control and cattle protection include vaccination of either species. A rabies vaccine for cattle was created in the early 1970's, but it is not widely used [13], [72]. Many ranchers do not vaccinate their cattle unless there was a recent rabies outbreak, even when *D. rotundus* are known to be in the area [1], [73]. Even though vaccination of all cattle is possible, the cost of routinely vaccinations can be prohibitive for smaller operations [10]. An oral vaccine can be mixed with vaseline and applied to the bat in the same manner as the “vampiricide gel”. The vaccine is also transferred to other bats in the roost. As bats groom themselves, they begin to develop immunity to rabies after ingestion [74]. The cost of this treatment method was analyzed with estimates from Massad et al. [3] and found to be cheaper than both the “vampiricide gel” and cattle vaccines [74]. Regardless of management strategy, our predictions help highlight areas that should receive priority.

Our results provide a current potential distribution of *D. rotundus* and can be used to indicate areas where cattle may be at an increased risk of being negatively affected by these bats. Additionally, our data can be compared with the published cattle density data set to locate areas with suitable habitat for cattle but not *D. rotundus*. Even though it can be hard to delineate these areas at such a large geographic scale, maps of smaller regions can easily be generated with a finer scale. We were able to predict potential change in distribution of the common vampire bat under three of the multiple climate change scenarios proposed. It is important to keep in mind these are simply predictions and are not indicative of a certain future. Our results will need to be re-assessed periodically when updated and more refined future climate data are available. Changes in land cover use could also affect our future predictions. Previous ecological changes from a natural to a more rural and agricultural landscape have favored *D. rotundus* expansion [33], [68], and this trend will likely continue in the future. The population in Latin America is projected to increase to 665 million by 2020 and the demand for livestock production will intensify [75]. To meet these demands, it is imperative the cattle industry minimize the negative impacts from *D. rotundus*. It is difficult to protect all cattle from the negative effects of *D. rotundus*, but we believe our risk map has significant implications for determining areas that would benefit most from rabies immunity.
